# Unique method for human villous trophoblasts isolation from placental tissue explants

**Published:** 2020-11-10

**Authors:** Ashley Serjilus, Donald J Alcendor

**Affiliations:** 1 Department of Obstetrics and Gynecology, School of Medicine, Meharry Medical College, USA; 2 Center for AIDS Health Disparities Research, Department of Microbiology, Immunology, and Physiology, Meharry Medical College, School of Medicine, Nashville, TN, USA

**Keywords:** placenta, trophoblasts, cytotrophoblasts, explants, isolation, cultivation

## Abstract

Isolation of cytotrophoblasts from primary placental tissue may be costly and time consuming with variable results. In this paper, we provide a simple, affordable, and efficient method that may performed using common laboratory supplies to achieve consistent *in vitro* isolation of cytotrophoblasts from villous tissue. Trophoblast populations are identified based on morphology and phenotyping, which employs the timely extraction of villous nodes from the placenta prior to cultivation and isolation of nodal outgrowth by visual guidance for selective capture of cytotrophoblast populations and subculture. This method allows for the isolation of cytotrophoblasts free of contamination with other placental cell types. Isolated cells stain positive for the specific cytotrophoblast biomarker cytokeratin 7 and Human Chorionic Gonadotropin (HCG). Subcultured cells grow to confluency to establish monolayers that may be passaged in culture and later used to develop primary syncytiotrophoblasts over time. These primary cytotrophoblast populations may be employed using in *in vitro* placenta-on-a chip models to better understand placental cell biology and function, as well as physiological responses after exposure to toxicants, and infectious agents. This technique may be modified for selective isolation of specific cell types within different tissues from multiple organ systems.

## Introduction

Conventional cell culture techniques that use different types of placenta-derived cells to understand placenta biology have been useful but lack organ-specific ultrastructure and physiological function of the placenta. Modeling of the human placenta to better understand placenta biology in human development has culminated into the establishment of placenta-on-a-chip models based on the compartmentalization of primary human placenta cells *in vitro* that can be monitored in real time [1–4]. These microdevices are created by using microfluidics and microfabrication technologies and microsystems consist of two polydimethylsiloxane (PDMS) microfluidic channels separated by a thin extracellular matrix (ECM) membrane [2]. Essential cellular components of these model placenta microsystems are placental trophoblasts.

The widespread use of commercially available trophoblast cell lines, such as BeWo, JEG-3, or JAR, that are derived from human choriocarcinoma, have been useful. However, these transformed cell lines, some of which have been in culture for decades, do not mimic primary trophoblasts *in vivo* [5–8]. The task of having to isolate primary cytotrophoblasts from placental tissue may be labor-intensive and require expensive reagents with variable results. Routine villous trophoblasts isolation based on trypsin digestion of placental villi, followed by additional purification steps, may be costly and labor-intensive [9]. Purification of villous trophoblasts using Percoll gradients was shown to yield a purity of about 80% by Kilman *et al.* [9–11]. Magnetic beads also have been used to further purify villous trophoblasts from trypsin- treated placental explants [12,13]. Other groups have demonstrated the separation of cytotrophoblasts from mononucleated syncytial fragments [14–16]. However, these procedures still required second-line separation technologies to achieve reasonable purity from other contaminating placental cell types. In this paper, we describe the procedure use to isolate purified villous cytotrophoblasts from term placental explants. This procedure involves the extraction of villous nodes from human placental tissue, continuous cultivation in trophoblasts media, and visual guidance for selective capture by clonal selection of cytotrophoblast populations, subculture, and validation of cytotrophoblast populations for identity and purity [17].

## Materials and methods

### Placenta collection

Placentas were obtained from elective non-laboring caesarean sections after uncomplicated full-term pregnancies at Vanderbilt University Medical Center. These studies were approved by the Vanderbilt University Institutional Review Board.

### Trophoblasts isolation

All procedures involved in the collection of placentas and placental explants and their transport were performed under sterile conditions. The time from delivery to placental node isolation did not exceed 1 hour. For cytotrophoblasts isolation, placental nodes or cotyledons were washed three times with phosphate buffered saline (PBS) pH7.4 and excised with sterile surgical scissors, and the decidual layer was removed to expose villous tissue. Placental nodes/cotyledons were minced with a number 21 scalpel, and individual 5-mm tissue explants were spatially added to 100×20-mm dishes and cultured in 10 mls of trophoblast medium per plate (10% fetal bovine serum, 1% penicillin/ streptomycin; ScienCell, Carlsbad, CA) [17]. Trophoblast medium was supplemented with 25 ug/ml of Fungizone (Gibco, Life Technologies, Grand Island, NY). Trophoblast explant outgrowth was examined daily by microscopy. Trophoblast colonies are identified using an inverted microscope. Distinct colonies were identified by morphology and encircled using a felt tip marker prior to extraction. Colony extraction was accomplished using the barrel of a plastic, sterile 1-ml pipette tip after the tip was removed with the scalpel. Media was removed from the dish, the open barrel of the pipette tip is dipped in sterile Vaseline, and the end of the pipette barrel containing Vaseline was placed directly over the trophoblast colony on the plate as the Vaseline created a seal around the cell colony. Trypsin is added (0.05% trypsin/EDTA (Gibco, Life Technologies, Grand Island, NY) to the open end of the pipette barrel for 10 min at 37°C. Trypsinized cytotrophoblasts were added to 4-well chamber slides containing trophoblast medium. Confluent cells were confirmed as trophoblasts by staining with the epithelial intermediate filament antigenic biomarker cytokeratin 7 (Millipore, Bedford, MA).

### Cells and viruses

The HCMV-GFP recombinant virus expressing green fluorescent protein was obtained from Dr. Gary Hayward, Johns Hopkins University. HCMV-GFP was cultivated in human foreskin fibroblasts at a multiplicity of infection (moi) of 01. Viral titers were determined by limiting dilution using a fluorescent focus assay [18]. All infections with the SBCMV clinical strain were performed at passage level 3 [18–19]. We are aware that high-level passage *in vitro* may result in the acquisition of mutations that may impact viral tropism and replication.

### HCMV-GFP infection of Placental histocultures

Approximately 20 grams of minced villous tissue were added to 30 ml of trophophoblast media in a 50-ml conical tube prewarmed to 37°C. The HCMV-GFP recombinant virus at a moi 0.1 was used to infect trophoblasts histocultures and was incubated for 1 hour at 37°C [17]. After 1- hour post-infection the media was removed, and fresh media was added. Next, the histocultures were incubated at 37°C for 96 hours. Infected villous tissue was placed in chamber slides and examine by fluorescent microscopy for GFP expression.

### Immunofluorescence

Immunohistochemistry was performed as previously published (Chamber slide cultures containing subcultured trophoblasts were washed twice with PBS (pH 7.4), air-dried, and fixed at -20°Cin absolute methanol for 10 minutes. Cells were air-dried for 15 minutes, hydrated in Tris-buffered saline (pH 7.4) for 5 min, and incubated separately for 1 hour with mouse monoclonal antibodies against cytokeratin 7, (Santa Cruz Biotechnology, Dallas TX), diluted 1:50 in PBS (pH 7.4). Cells were washed three times with Tris saline and then incubated at 37°C for 30 minutes with a combination of secondary donkey anti-mouse Immunoglobulin G (IgG) antibodies, conjugated with fluroescein isothiocyanate (FITC), (Jackson ImmunoResearch, West Grove, PA) at a 1:100 dilution in PBS [18,19] . Cells were washed another three times in Tris saline and mounted with Vectashield mounting media (Vector Laboratories, Burlingame, CA) containing 1.5 μg/ml of 4’,6-diamidino-2-phenylindole (DAPI). Fluorescence was photographed with a Nikon TE 2000 S fluorescent microscope mounted with a charge-coupled device (CCD) camera (Nikon, Tokyo, Japan).

### Immunohistochemistry

Primary trophoblasts were cultivated in chamber slides at a density of 1×10^4^ cells/well in trophoblasts media with and without forskolin for 72 hours. Cells were wash 3X with PBS pH 7.4, air dried at room temperature, and fixed in 100% methanol at -20C for 30 minutes. Cells were then air dried, hydrated in PBS, and immunohistochemistry (IHC) was performed as previously described [19] using a mouse monoclonal antibody to Human chorionic gonadotropin (HCG). DAB was used as a peroxidase substrate for color development. Positive trophoblasts appear brown in color.

### Forskolin treatment

Primary trophoblasts were cultivated in chamber slides at a density of 1×104 cells/well in trophoblasts media and treated with 25 μM of forskolin (Sigma-Aldrich Inc.) for 72 hours. Mock treated cells were given media only.

## Results

### Explant cultivation

**Figure 1.**
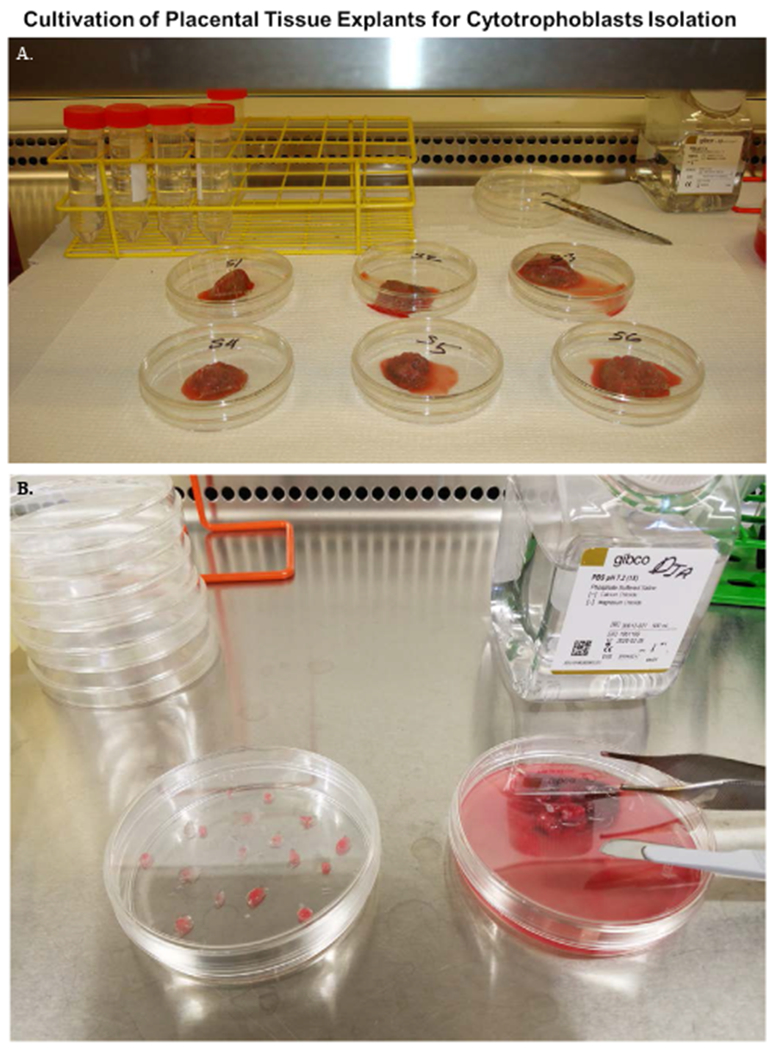
Cultivation of placenta explants for cytotrophoblast isolation. Villous nodes/cotyledons are excised from placentas with a scalpel and surgical scissors in a laminar flow hood and transported on ice in 50 ml conical tubes containing 30 ml trophoblasts media. Nodes are soaked in access PBS pH7.4 prior to dissection and cultivation

Nodes are collected from intact placentas under sterile conditions. Villous nodes were first submerged in complete trophoblast media and transported on ice. Before dissection, nodes were washed in an excess of PBS to reduce erythrocyte contamination and hydrate the tissue (Figure 1). Villous nodes are sectioned with a scalpel and surgical scissors and placed in dishes. Fresh, prewarmed trophoblast media was added, and explant cultures were incubated at 37C for 1 -16 days, with fresh media changes every 2 days.

### Placental histocultures support HCMV-GFP replication

**Figure 2.**
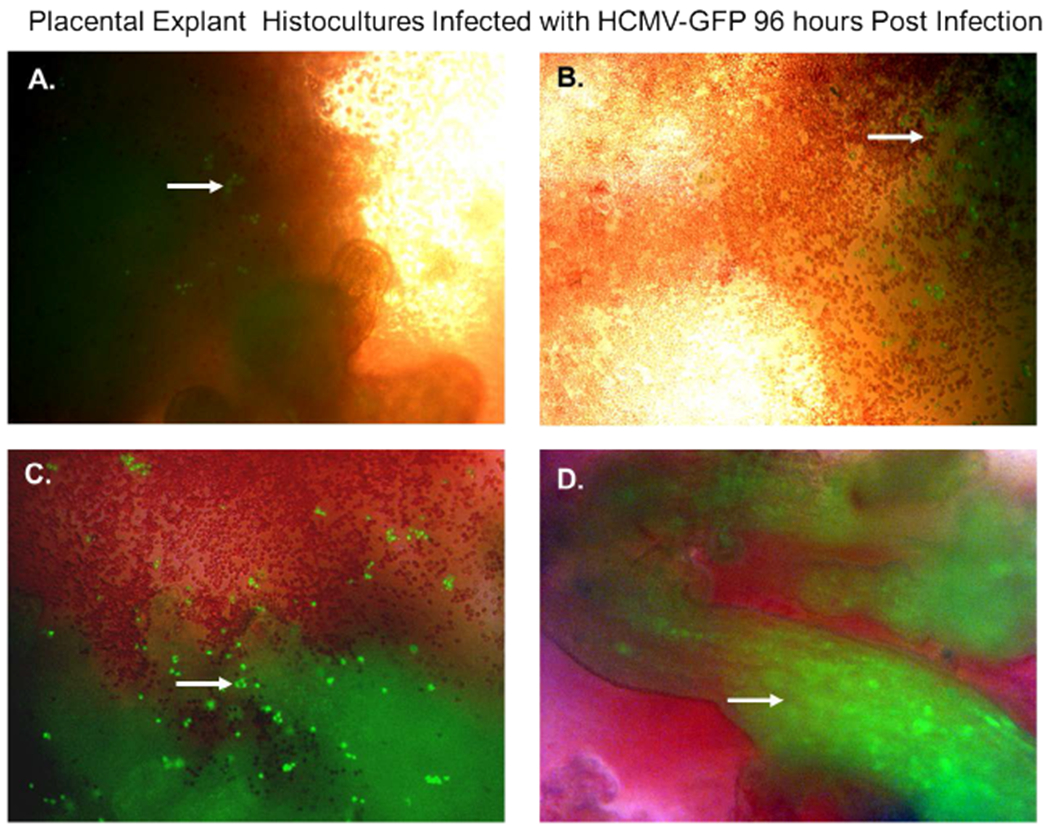
Placental explant histocultures infected with HCMV-GFP Placental explant histocultures were infected with HCMV-GFP and examined for GFP fluorescence 96 hours postinfection. (**A**). Fluorescent microscopy of viable cells infected with HCMV-GFP expressing green fluorescent protein in peripheral placental tissue as shown by the white arrow. (**B**). Fluorescent microscopy of viable cells infected with HCMV-GFP expressing green fluorescent protein in peripheral placental tissue and contamination with erythrocytes as shown by the white arrow. (**C**). Fluorescent microscopy of viable cells infected with HCMV-GFP expressing green fluorescent protein in aggregate issue and contamination with erythrocytes as shown by the white arrow. (**D**). Fluorescent microscopy of viable cells in villous tree structures infected HCMV-GFP expressing green fluorescent protein as shown by the white arrow. All images were obtained using a Nikon TE2000S microscope mounted with a charge-coupled device (CCD) camera at ×200 magnification

In a previous publication, we determined that villous trophoblasts are highly permissive for HCMV lytic replication. To determine if there were viable cells that could support replication of HCMV, we infected histocultures of villous nodes with a recombinant HCMV-GFP. Villous nodes are known to be enriched with villous cytotrophoblasts, and knowing their permissiveness for HCMV, we would expect GFP expression only in cells that could support HCMV replication (Figure 2). At 96 hours post-infection, we observed GFP-positive cells by fluorescent microscopy (Figure 2) in peripheral tissue, isolated cells (Figure 2A, 2B, and 2C), and internal villous tree structures (Figure 2D). We also observed a significant amount of erythrocyte contamination in all specimens examined (Figure 2).

### Nodal outgrowth of cytotrophoblasts

**Figure 3.**
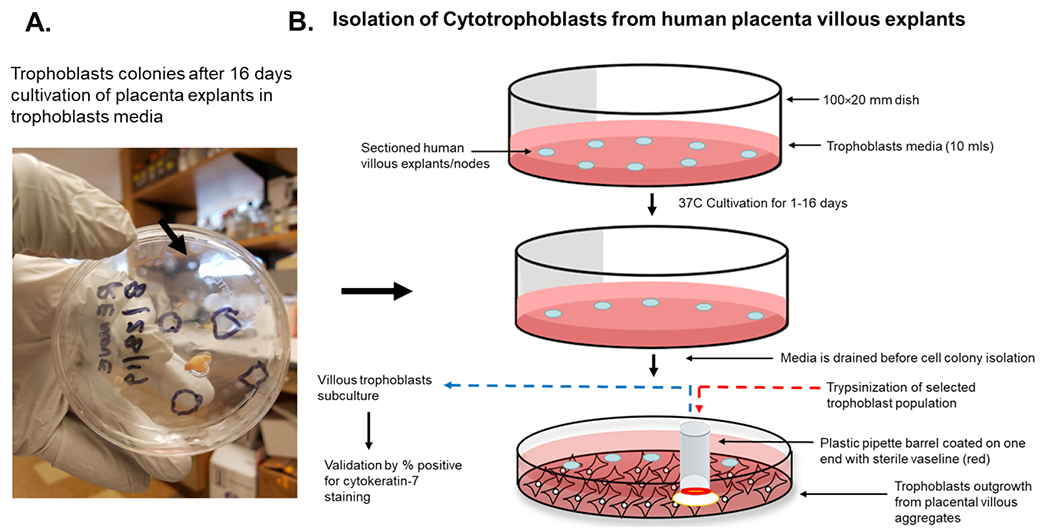
Scheme for isolation of cytotrophoblasts from placental explants. The isolation of trophoblasts involves the sectioning and placement of villous explants on a petri dish and cultivation and isolation of selected cell colonies from outgrowth from villous aggregates using a simple pipette barrel coated with Vaseline, followed by tiypsinization and subculture to access purity by cytokeratin 7 staining

**Figure 4.**
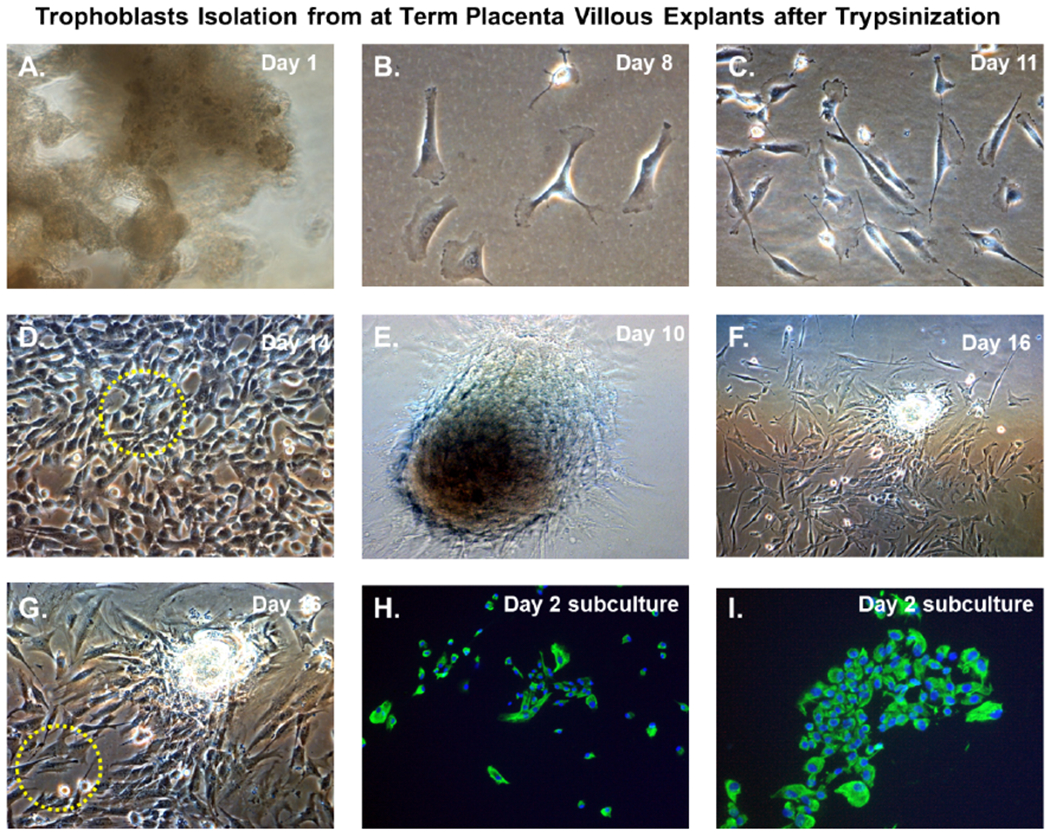
Trophoblasts isolation from at-term placenta villous explants after trypsinization. Time course isolation of trophoblasts from at-term placenta villous explants after trypsinization **(A)** placental nodes/cotyledons 24 hours after excision and dissection cultivated in trophoblasts media; **(B)** Villous cell outgrowth and early colony formation 8 days post-cultivation; **(C)** Villous cell outgrowth and subconfluent colony formation 11 days post-cultivation; (**D**) Villous cell outgrowth and confluent focal monolayers 14 days post-cultivation with yellow-dotted outlined areas designated for clonal selection and expansion; **(E)** Villous tissue aggregates in culture at 10 post-cultivation **(F)** Villous tissue aggregates and associated villous cell outgrowth 16 days post-cultivation **(G)** Higher magnification of villous tissue aggregates and associated villous cell outgrowth 16 days post-cultivation with yellow dotted outlined areas designated for clonal selection and expansion; **(H)** Subculture and cultivation of clonally selected cells that stain positive for cytokeratin 7 by immunofluorescent staining after 24 hours; **(I)** Subculture and cultivation of clonally selected cells that stain positive for cytokeratin 7 by immunofluorescent staining after 48 hours. For immunostained images, nuclei were stained blue with 4’,6-diamidino-2-phenylindole (DAPI). All images were obtained using a Nikon TE2000S microscope mounted with a charge-coupled device (CCD) camera at ×200 magnification

An illustration of the cultivation process for isolation of trophoblasts is shown in Figure 3. We cultured villous explants in 100×20 mm dishes in 10 ml of trophoblast medium after trypsinization for isolation of villous trophoblasts over the course 16 days (Figure 3). Fresh media was added every 2 days during the cultivation period. On day 1, explants appeared aggregated, and some were adherent (Figure 4A). At 8 days post-cultivation I observed small colonies of cells with characteristic trophoblast morphology (Figure 4B). On day 11, we observed a significant expansion of these colonies that remained subconfluent (Figure 4C). After 14 days, we observed patches of confluent growth with trophoblasts morphology (Figure 4D). These cells were round or oval, or was multipolar, elongated cell monolayer previously described as “crazy pavement” [20–23]. In addition, we observed what appeared as trophoblasts aggregates 10 days post-cultivation (Figure 4E). At day 16, we observed significant cell outgrowth from these villous trophoblasts aggregates (Figure 4F). After examination, we observed that these cells had features consistent with villous trophoblasts.

### Selective capture and subculture of purified cytotrophoblast populations

Nodal outgrowth of satellite cells that developed into confluent mini-colonies, which became subconfluent over time (Figure 4D), and cell outgrowth obtained from nodal aggregates (Figures 4F and 4G) were preferred for selective capture and subculture. These colonies appeared more homogenous and had morphological characteristics and growth rates consistent with trophoblasts monolayers. Several colonies were selected using the trypsin-pipette-Vaseline method of isolation, as described in the Materials and Methods section (Figure 3). Trypsinized cells from colonies were subcultured in duplicate chamber slides for both cell expansion and validation by immunofluorescent staining (Figure 3).

### Validation of trophoblast isolation

**Figure 5.**
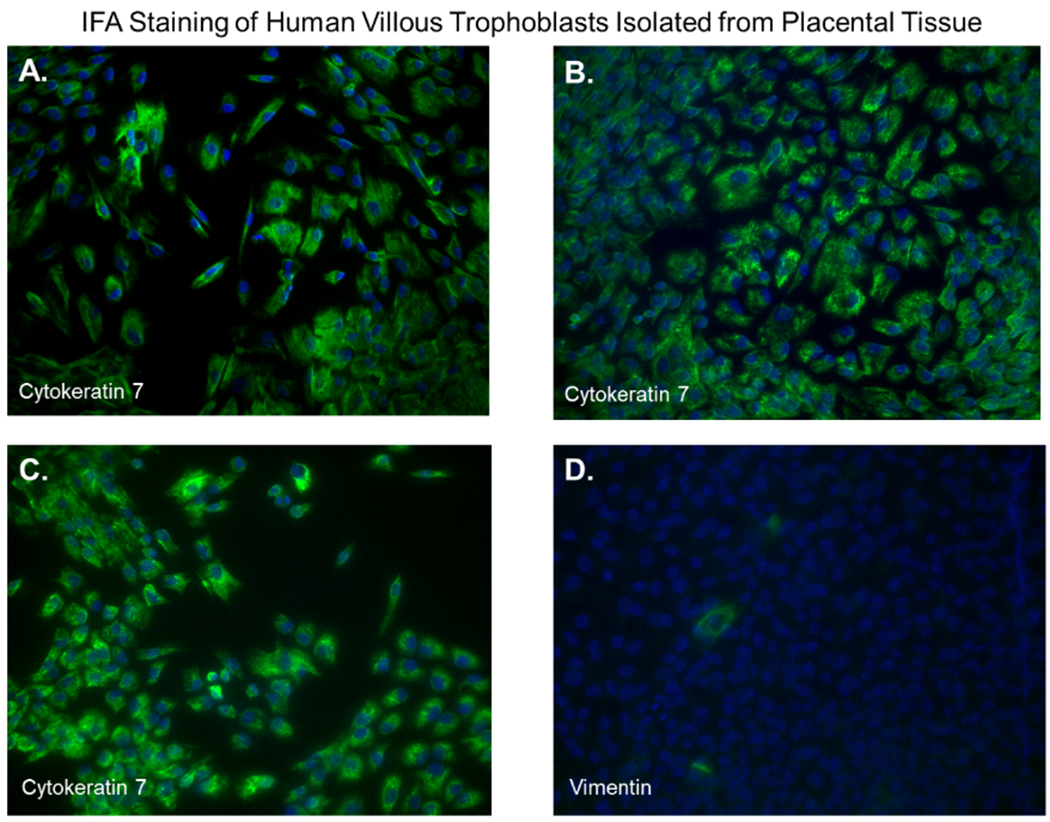
Immunofluorescent staining of purified trophoblasts expressing cytokeratin-7. Subcultured cytotrophoblasts are expanded in culture for 96 hours in chamber slides. **(A-D);** Cells stained by immunofluorescent staining with antibodies against cytokeratin 7. Nuclei were stained blue with 4’,6-diamidino-2-phenylindole (DAPI). All images were obtained using a Nikon TE2000S microscope mounted with a charge-coupled device (CCD) camera at ×200 magnification

**Figure 6.**
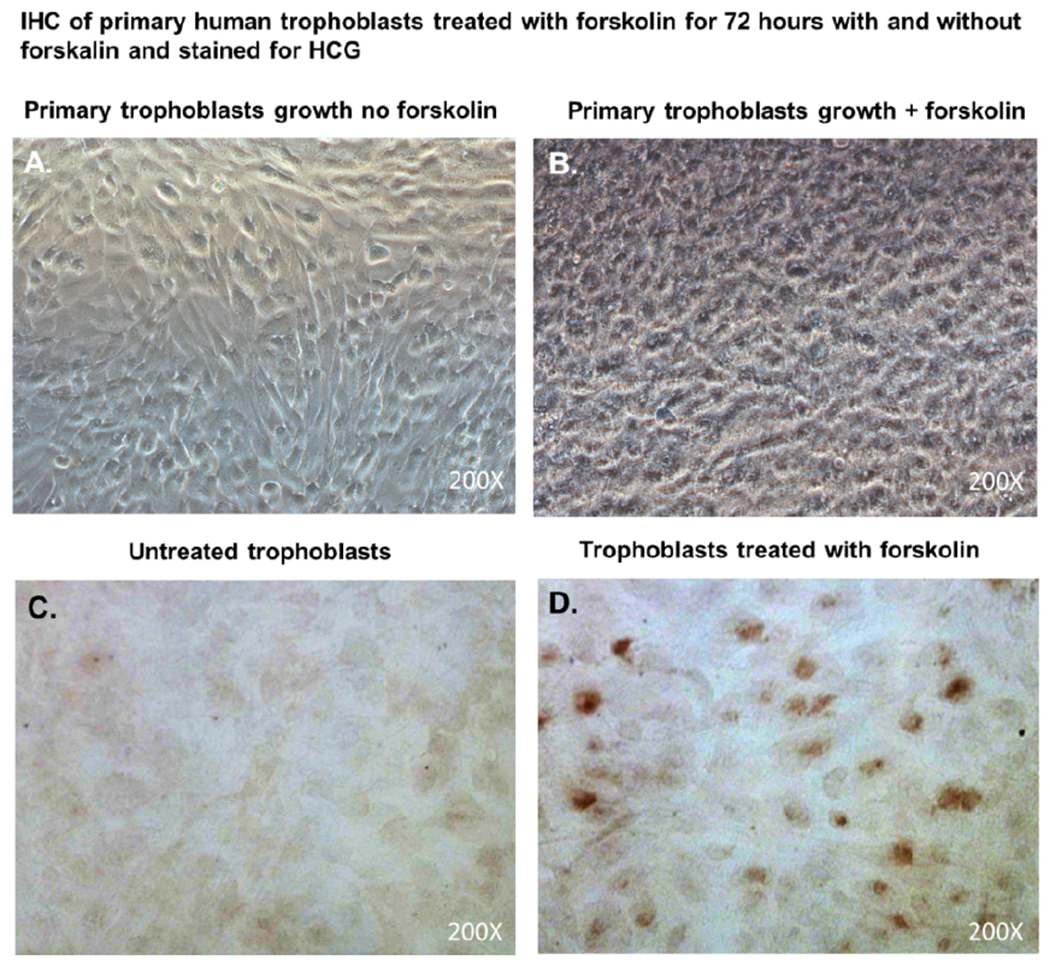
Cytotrophoblasts to syncytiotrophoblast transition and Immunhistochemical staining for HCG. Subcultured cytotrophoblasts are expanded in culture for 72 hours in chamber slides to accomplish cytotrophoblast to syncytiotrophoblast transition; (**A**); Primary trophoblasts cultivated in chamber slides for 72 hours without forskolin; (**B**) Primary trophoblasts cultivated for 72 hours with forskolin;(**C**) Primary trophoblasts cultivated for 72 hours without forskolin and were stained immunohistochemistry with antibodies against Human HCG; (**D**) Primary trophoblasts cultivated for 72 hours without forskolin and were stained immunohistochemistry with antibodies against Human chorionic gonadotropin (HCG). DAB was used as a peroxidase substrate for color development. Positive trophoblasts appear brown in color. All images were obtained using a Nikon TE2000S microscope mounted with a charge-coupled device (CCD) camera at ×200 magnification

After 48 hours, we observed that subcultured, subconfluent cell populations were viable, adherent, and stained positive for the trophoblast antigenic biomarker cytokertin 7 (Figure 4H and 4I). After cells became confluent, I observed universal cytokeratin 7 staining of purified cytotrophoblasts by fluorescent microscopy (Figures 5A–5D). Primary trophoblasts were cultivated in chamber slides at a density of 1×104 cells/well in trophoblasts media with and without forskolin for 72 hours. Cells treated forskolin had higher levels of HCG as determined by IHC staining (Figures 6A–6D).

## Discussions

Several methods exist for isolating trophoblasts from placental tissue. In this study, we explore the cultivation of villous trophoblasts from placental nodes/cotyledons. The trypsin-pipette-Vaseline method includes Vaseline to seal the edges of the pipette barrel for focal trypinization. Creating the seal using Vaseline prevent crosscontamination by floating cells or cells from distal colonies that can occur when using cloning cylinders (Figure 2) [24]. We are aware of the similarities that exist among the different methods for isolation of trophoblast populations involving standard trypsinization; however, we employed a simple, affordable, and efficient for method that may be performed with common laboratory supplies to achieve consistent *in vitro* isolation of purified trophoblasts from placental villous tissue. Selected colonies are first identified during early stages of colony formation as shown by figure 4B. The colonies when identified are followed in development by microscopy until it reaches visual size for selection. The selection process only continues if the subcultured cells stain positive for the universal trophoblasts marker cytokeratin-7 and not for vimentin (mesenchymal marker) as observed in Figure 5. If trophoblasts purification techniques require tissue homogenization this will increase the likelihood endothelial cell contamination. Our procedure for trophoblast isolation does not involve tissue homogenization. This method relies of phenotyping with cytokeratin 7 and vimentin to gauge purity. Maldonado-Estrada et al., evaluated the use of Cytokeratin 7 as an accurate intracellular marker to assess the purity of human placental villous trophoblast cells by flow cytometry [25]. They demonstrate that the mutually exclusive pattern of intracellular CK7/vimentin expression of human trophoblast may be used for evaluation by flow cytometry of the purity of primary human trophoblast cells post-isolation [25]. Identification was also supported HCG expression in primary trophoblasts after treatment with Forskolin [26] (Figure 6). Several techniques have been evaluated for the isolation and purification of placental cell types mainly focused on mesenchymal stromal cells isolated from various parts of the placenta or epithelial cells isolated from amniotic membrane including cytotrophoblasts [27]. Our technique may be modified for selective isolation of cell populations from a variety of different tissues. In addition, we have used this same procedure for isolation and purification of placental fibroblasts [17]. Using this approach with multiple term placentas we have obtained reliable results. However, cultivation and yield can vary greatly if placental tissue is delayed in processing before the subculture of primary explants are performed. An important limitation to this procedure is that the initial isolation of these colonies requires extensive cultivation in vitro however, colonies will continue to develop over time (3-5 cells in the initial colony as seen in Figure 4B) and you can continue to select colonies as needed from the same placental tissue. This method is cost effective due the total amount of time a technician needs to interact with the raw tissue and the cells during cultivation. The greatest cost overall is the technicians’ time and consumables.

## Conclusions

In this study, I described a unique method for the isolation of cytotrophoblasts from term placenta tissue explants. This method allows for routine cultivation and purification of villous trophoblasts from placental tissue explants. Limitations exist with this approach, namely the time required for placental trophoblast outgrowth may vary and the rare possibility of low-level contamination with other placental cell types. This method will depend on freezing and storage of purified trophoblast populations to avoid synciatialization of cytotrophoblasts in culture over time.
